# Depression, anxiety, and stress at different time frames in relation to COVID-19 disease course: a Flemish observational study

**DOI:** 10.3389/fpsyg.2026.1765017

**Published:** 2026-10-20

**Authors:** Gwendy Darras, Mattias Desmet, Wim Schoutens, Dries Debeer, Andrea E. Lamont, Jayanta Bhattacharya, Lieven Annemans

**Affiliations:** 1 Department of Psychoanalysis and Clinical Consulting, Ghent University, Ghent, Belgium; 2 Department of Statistics and Risk, University of Leuven, Leuven, Belgium; 3 Department of Experimental Psychology, Ghent University, Ghent, Belgium; 4 Independent Researcher, Lexington, SC, United States; 5 Department of Health Policy, Stanford University School of Medicine, Stanford, CA, United States; 6 Department of Public Health and Primary Care, Ghent University, Ghent, Belgium

**Keywords:** anxiety, COVID-19, depression, disease course, psychological distress, SARS-CoV-2 infection, stress

## Abstract

**Introduction:**

Psychological distress can impact the course of viral infections such as COVID-19. This study aimed to examine how depression, anxiety, and stress, which occurred before and during COVID-19 infection, are associated with COVID-19 disease course (number of symptoms and duration of symptoms) in Flemish outpatients.

**Methods:**

In early 2022, a two-wave web-based survey was administered to 3,025 adults who had a positive COVID-19 PCR test. Psychological distress was assessed using the Depression, Anxiety, and Stress Scale-21 (DASS-21) and COVID-19 disease course with a set of self-constructed items. Generalized linear models were used to evaluate the associations. Several control variables were included in the analysis, such as vaccination status, comorbidities, and previous SARS-CoV-2 infection.

**Results:**

Across all time frames, psychological distress showed consistent, statistically significant associations with both aspects of the COVID-19 disease course (with the exception of psychopharmaceutical use 3 years before COVID-19 infection and duration of symptoms), albeit with small to moderate effect sizes typical of psychological research. Across all time frames, anxiety during infection showed the largest point estimates with number of symptoms (IRR: 1.20, 95% CI 1.16 to 1.23) and duration of symptoms (OR: 1.79, 95% CI 1.55 to 2.07). However, there was no statistical evidence that these associations differed in magnitude from other types of distress. When focusing on psychological distress before COVID-19 infection, anxiety short-term before infection had the largest point estimate for number of symptoms (IRR: 1.13, 95% CI 1.09 to 1.16) and duration of symptoms (OR: 1.40, 95% CI 1.22 to 1.61), although we did not find evidence of differences in effects across types of distress.

**Conclusion:**

All measures of psychological distress were significantly associated with worse COVID-19 outcomes, except for the association between psychopharmaceutical use 3 years before COVID-19 infection and duration of symptoms. The magnitude of effects showed a consistent trend whereby anxiety short-term before and during COVID-19 infection showing the strongest associations, though not significantly different. This observational survey study has a few notable limitations associated with measurement and casual inferences, including those related to recall bias and self-report of both independent and outcome variables. Further research is needed to clarify these associations in a causal and mechanistic framework.

## 1 Introduction

Before the COVID-19 pandemic, depression and anxiety were the most common mental health problems in the general population and leading contributors to the global burden of disease (GBD 2019 Mental Disorders Collaborators, 2022; Prince et al., 2007; Şahan et al., 2021). In 2016, one in six people in the EU had a mental health diagnosis (Scholz, 2021). During the COVID-19 pandemic, global mental health deteriorated, manifesting in increased loneliness, depression, anxiety, worry, insomnia, harmful alcohol and drug use, self-harm, and suicidal behavior (see, e.g., Pathirathna et al., 2022; Vindegaard and Benros, 2020). Furthermore, the number of diagnosed cases of mental disorders rose dramatically, with an additional 76.2 million cases of anxiety and 53.2 million cases of major depressive disorders worldwide (COVID-19 Mental Disorders Collaborators, 2021). The World Health Organization (2022) estimated that the first year of the COVID-19 pandemic led to a 25 percent increase in anxiety and depression worldwide.

In addition to the fear of the virus itself, lockdowns and quarantine measures posed a significant burden on global mental health (Jones et al., 2023). These measures brought about major changes to daily routines, living and working conditions, and the social and economic prospects of the world’s population. Depending on the personal situation of citizens, this might impact their psychological well-being. Individual responses to the COVID-19 crisis varied: some experienced increased psychological distress, others remained stable and some even reported improved well-being (Shevlin et al., 2023). The psychological impact of both the threat of the virus and of the measures might have interacted in a complex way with pre-existing feelings of stress (Şahan et al., 2021; Vindegaard and Benros, 2020).

While much attention has been paid to physical conditions that exacerbated COVID-19 disease course (e.g., obesity and age), far less attention has been given to the impact of pre-existing mental health on physical outcomes. Psychological distress is conceptualized as a broader construct reflected by related but distinct dimensions of depression, anxiety, and stress (Kompaniyets et al., 2021). Symptoms of depression (e.g., dysphoria, hopelessness, anhedonia) may impede healing in ways that exacerbate disease. Similarly, anxiety (e.g., autonomic arousal, anxious affect) and stress (e.g., difficulty relaxing, nervous arousal) may interfere with parasympathetic activation required for recovery. However, the extent to which pre-existing and concurrent psychological distress influenced individual health outcomes during the pandemic remains unclear. This study aimed to examine how depression, anxiety, and stress, reflecting periods before and during COVID-19 infection, are associated with disease course.

### 1.1 Psychological distress and immune functioning

Psychoneuroimmunology (the study of the interaction between mental processes, neural function, endocrine function, and immune responses) provides a framework for understanding how psychological processes influence immune function and health (Ader, 2000; Cohen et al., 1991; Cohen, 2021; Cohen et al., 2007; Bower and Kuhlman, 2023; Bower et al, 2022). Historically, inflammation and infectious disease were conceptualized primarily as the result of genetic and biologic processes in the presence of environmental pathogens. Recently, empirical evidence increasingly points to the role of psychological factors and biopsychosocial mechanisms on immune functioning and physical health (e.g., Alotiby, 2024; Vasile, 2020). A meta-analysis of over 300 empirical studies concluded that as a stressor becomes more chronic, an increasing number of components of the immune system are adversely affected (Segerstrom and Miller, 2004). Mechanistic studies demonstrate how chronic stress may modulate immune functioning in complex and multifactorial ways, including increased inflammation, dysregulation of the hypothalamic–pituitary–adrenal (HPA) axis and nervous system, and changes in the microbiome (Marwaha et al., 2025; Warren et al., 2024; Morys et al., 2024; Alotiby, 2024; Nunez et al., 2025). These processes likely contribute to heterogeneity in health outcomes and variability in disease progression observed in clinical settings. Understanding biopsychosocial pathways to health is a critical step for elucidating disease pathogenesis and identifying modifiable targets for intervention.

Psychological distress has been shown to negatively affect the disease course of viral infections, specifically viral respiratory infections, and contribute to adverse physical health outcomes (see, e.g., Goodwin et al., 2014; Falagas et al., 2010; O’Connor et al., 2021, Cohen, 2021; Cohen et al., 2007; Cohen et al., 1991, Graham et al., 2006; Segerstrom and Miller, 2004). Epidemiological studies indicate that psychological distress may have played a role in the disease course of SARS-CoV-2 infection, specifically. For example, a study of 1,847 Polish outpatients indicates that psychological distress as well as overwork and night shifts before the SARS-CoV-2 infection significantly worsened the severity of acute COVID-19 infection (Pływaczewska-Jakubowska et al., 2022). Kim et al. (2021) and Ayling et al. (2022) reported similar results, demonstrating that sleep problems and psychological distress before or at the onset of the pandemic increased the severity of COVID-19 disease. Koyama et al. (2022) studied a database of more than 650,000 hospitalized COVID-19 patients, and found that mental health conditions such as anxiety, depression, bipolar disease and schizophrenia were associated with increased risk for same-hospital readmission and longer length of stay. Anxiety was also associated with an increased risk of intensive care unit admission, invasive mechanical ventilation, and death. Molero et al. (2023) conducted a meta-analysis of 81 studies that tested the relationship between mental illness and SARS-CoV-2. On average, across studies, the authors did not find support that pre-existing, diagnosable mental disorders increased the likelihood of acquiring SARS-CoV-2, however, both morbidity and mortality rates, the need for care related to COVID-19 once infected, and long COVID-19 symptoms were all related to pre-existing mental conditions.

Early data on psychoneuroimmunological mechanisms in COVID-19 suggest involvement of a stress–immune (psychoneuroimmunological) pathway, mediated in part by HPA axis activation, in the transmission of risk from psychological disturbance to immune dysfunction and SARS-CoV-2 infection. A small study of 30 hospitalized patients found that those with more severe COVID-19 reported greater lifetime stress and exhibited higher cortisol levels compared to those with less severe disease (Ramezani et al., 2020), implicating the role of stress and anxiety on the vulnerability to infection. In this study, patients with fatal outcomes showed higher serium cortisol levels compared to those who survived (Ramezani et al., 2020). This finding was replicated in subsequent studies, emphasizing the importance of psychoneuroimmunological stress–immune pathways in the manifestation of COVID-19, especially in severe cases (see, e.g., Afrisham et al., 2023; Amiri-Dashatan et al., 2022).

This short review indicates that these factors extend beyond physical health and suggest an association between mental health and COVID-19 disease course. But it also identifies some gaps in the corpus of evidence. Most studies investigated only a segment of the general population, such as hospitalized COVID-19 patients, healthcare workers, psychiatric patients, and certain age groups, such as students or middle-aged people. Although most COVID-19 patients experienced the disease at home, to our knowledge, only three small-scale studies investigated this population, one with non-confirmed COVID-19 cases. Furthermore, none of the studies included all relevant control variables such as vaccination status, comorbidities, and body mass index (BMI).

Finally, most studies did not differentiate between different types of psychological distress, nor did they distinguish psychological distress occurring long-term before, medium-term before, short-term before and during the COVID-19 infection. These differentiations may provide a more nuanced understanding of the relationship between psychological distress and the COVID-19 disease course.

### 1.2 The current study

The current study aims to further the literature on psychoneuroimmunology in COVID-19 response. Specifically, we aim to (1) examine the associations between different types of psychological distress and the COVID-19 disease course, and subsequently (2) assess which type of psychological distress (anxiety, depression, stress) is most strongly associated with adverse COVID-19 outcomes (number of symptoms and duration of symptoms), and (3) evaluate whether the time frame (long-term before, medium-term before, short-term before, and during COVID-19) of psychological distress might influence these outcomes in a sizeable non-hospitalized sample of the general Flemish population, including relevant control variables.

Based on the literature, we expect (1) significant relationships between the different types of psychological distress and adverse COVID-19 outcomes. Furthermore, (2) we cannot formulate a specific hypothesis regarding which type of psychological distress is most strongly associated with adverse COVID-19 outcomes, as the literature does not provide a clear foundation. Additionally, we expect that (3) the duration of the psychological distress will be positively associated with the severity of the COVID-19 disease course.

## 2 Methods

### 2.1 Study design and procedures

In this retrospective, observational study, we used a two-wave online survey to collect data on psychological distress (depression, anxiety, stress) and COVID-19 outcomes: The first survey was completed immediately after respondents were informed that they tested positive for COVID-19. A second online survey was conducted 3 weeks later. Eligibility criteria specified that participants should be Dutch-speaking, aged 18 or older and had taken a positive PCR test for COVID-19 in the past week. Because the Flemish government largely reimbursed PCR testing for COVID-19 through health insurance, we were also able to include disadvantaged segments of the Flemish society. Both online surveys (Limesurvey v3.15) could be completed on a computer or on a mobile device. A special webpage was created for the study with an online registration form where people could sign up to participate.

Recruitment took place in three different ways. At the COVID-19 testing center of Ghent University Hospital, posters were displayed, and a volunteer distributed flyers to adults undergoing PCR testing for COVID-19. Secondly, adults with a positive test result received a text message from the University Hospital of Ghent inviting them to participate in the study. Thirdly, we collaborated with the Agency for Care and Welfare of the Flemish government, responsible for COVID-19 contact tracing. The contact tracers invited Flemish adults who had a positive COVID-19 PCR test to participate in the study. Before entering the study, informed consent was obtained from all participants electronically. The Ethics Committee of Ghent University Hospital approved this study’s procedures (No. BC-11395).

Data collection occurred between February 9 and June 5, 2022. On the research webpage, 5,572 people signed up to participate in the study, of which 4,779 participants started by completing the first survey. 4,431 participants answered all questions. Survey two was started by 3,309 participants, of which 3,156 completed the entire survey. 131 participants were excluded from the final dataset as they provided incomplete or impossible data (e.g., an impossible value for length or weight).

### 2.2 Measures

#### 2.2.1 Psychological distress at different time frames

*Psychological distress long-term before COVID-19* was assessed through two self-constructed binary questions: ‘Have you been unable to work in the past three years due to mental health problems, such as burnout, depression, anxiety attacks, etc.?’ or ‘Have you been treated with psychopharmaceuticals during the past three years?’ A positive response to either or both questions was considered indicative of long-term psychological distress. These binary items were included as complements to the DASS-21 (see below) to capture discrete, salient events (e.g., work impairment, pharmacological treatment), which are typically more reliably recalled than subjective psychological distress levels.

*Depression, anxiety, and stress medium-term before, short-term before, and during COVID-19* were assessed through the standardized psychological survey Depression, Anxiety, and Stress Scale-21 (DASS-21), a shortened version of the DASS-42, designed to measure emotional states of depression, anxiety, and stress (Lovibond and Lovibond, 1995). The validated DASS-21 consists of 3 subscales, each comprising seven items, scored on a four-point scale from *never* (0) to *almost always* (3). Higher subscale scores reflect higher levels of depression, anxiety, and stress, with a higher total score representing greater general psychological distress. The scales of the validated Dutch version exhibit a good internal consistency, with Cronbach’s alpha values ranging from 0.75 to 0.95, and demonstrate strong construct and criterion validity (Wardenaar et al., 2018). We used the scale three times. First, as part of the first survey, as a measure of psychological distress short-term before (1 week) COVID-19 infection by using the time indication ‘last week’ as prescribed by Lovibond and Lovibond (1995). Second, also as part of the first survey, it is used to assess the emotional states medium-term before (1 year) the COVID-19 infection by using ‘last year’ as a time indication. Third, as part of the second survey, we adapted the time indication to ‘the past three weeks’ to measure psychological distress during the COVID-19 disease course. We acknowledge that these deviations from the original instrument’s time anchors may affect its psychometric properties; however, no validated instruments using the same items across varying time frames were identified, necessitating this adaptation for the purposes of the study.

#### 2.2.2 COVID-19 disease course

We assessed two COVID-19 outcomes to measure the severity of the participants’ COVID-19 disease course.

##### 2.2.2.1 Number of COVID-19 symptoms

A checklist of 15 self-reported symptoms (fever, shortness of breath, chest pain, headache, muscle aches, sore throat, diarrhea, stuffy nose or mucus in the throat, coughing, joint pain, fatigue or malaise, loss of taste or smell, irritated eyes, impaired speech or movement, and altered consciousness or confusion) was used to determine the number of symptoms.

##### 2.2.2.2 Duration of COVID-19 symptoms

Participants were divided into two groups, using a cut-off point at 5 days, which was selected based on relevance from a medical point of view. The five-day mark represents a clinically meaningful distinction between shorter illness and more prolonged symptom duration, where more active treatment may be required. The analyses using alternative cut-off points (i.e., 0 and 10 days) are presented in the Supplementary materials.

#### 2.2.3 Control variables

We included the following control variables in our analyses:

##### 2.2.3.1 Vaccination status

COVID-19 vaccination can lower the risk of severe illness (Martínez-Baz et al., 2023). At the moment of the data collection approximately 85 percent of the Flemish population was vaccinated (Sciensano, 2022). We assessed participants’ vaccination status against SARS-CoV-2 using a yes-or-no question.

##### 2.2.3.2 Previous infection

Previous COVID-19 infection has been shown to mitigate the severity of subsequent infections (Netea and Li, 2021). Therefore, we assessed whether the participant had previously tested positive for COVID-19, using a yes-or-no question.

##### 2.2.3.3 Comorbidities

Several underlying medical conditions are known to have a negative impact on COVID-19 disease course (Centers for Disease Control and Prevention, 2023). We included asthma, chronic kidney disease, cerebrovascular disease, chronic obstructive pulmonary diseases, chronic liver diseases, diabetes mellitus type 1 and 2, and obesity. The total number of comorbidities was used as a continuous variable.

##### 2.2.3.4 Body mass index

The risk for adverse COVID-19 outcomes grows for every BMI unit increase. A BMI score of 20 kg/m2 or less could lead to severe COVID-19 illness as well (Gao et al., 2021). For this item we asked for the length and weight of the respondents and calculated the corresponding BMI score per individual, used as a continuous variable.

##### 2.2.3.5 Use of corticosteroids or immunosuppressive medications

The use of corticosteroids or immunosuppressive medications can impair immune system function (Cain and Cidlowski, 2017). We asked participants whether they had used one of these medications in the 3 months prior to infection. Responses were recoded into a binary variable.

##### 2.2.3.6 Gender

Studies show that men experience both a higher severity and fatality for COVID-19 infection than women (Scully et al., 2020). Gender was used as a binary variable.

##### 2.2.3.7 Age

According to the Centers for Disease Control and Prevention (2023) age remains a strong risk factor for severe COVID-19 outcomes, with risks increasing with age. Age was used as a continuous variable.

##### 2.2.3.8 Educational level

Socioeconomic status, which is associated with poorer health outcomes, such as a more severe COVID-19 disease course (Wachtler et al., 2020), was represented by educational level, categorized into three groups according to the International Standard Classification of Education: low educated (from early childhood education to lower secondary education), medium educated (upper secondary education to post-secondary non-tertiary education), and high educated (short-cycle tertiary education to doctoral degree or equivalent; Eurostat, 2022).

##### 2.2.3.9 Smoking

Smoking is a risk factor for severe COVID-19 outcomes and was assessed with a yes-or-no question (Patanavanich and Glantz, 2020).

##### 2.2.3.10 Use of vitamins and dietary supplements

Nutrition is intricately linked to immunity, as well as the risk and severity of infections. Epidemiological studies show that individuals who are poorly nourished are at greater risk of adverse clinical outcomes, as the body’s immune response depends on the presence of essential micronutrients (Calder, 2021). This item assesses regular use of vitamins and dietary supplements, recorded as a yes-or-no variable.

### 2.3 Statistical analysis

The statistical analyses were performed using the R software (version 4.3.3). First, descriptive statistics of all measures were calculated: means and standard deviations for the continuous variables and frequencies and proportions for the categorical variables. Second, all continuous predictors were standardized before analysis. Binary categorical variables were contrast-coded in all models, with 0.5 weights. Third, generalized linear models were fitted for each outcome variable. Due to the high correlation between the various psychological distress variables, and corresponding high Variance Inflation Factor (VIF) values indicating multicollinearity, we ran a series of models that took this collinearity into account. First, we ran each timeframe with the sum score of psychological distress; then, we ran separate models for each measure of psychological distress in a particular time frame as a predictor variable and 10 control variables. Hence, the association of each distress measure on top of the control variables was investigated. More specifically, in the analyses of the outcome variable number of COVID-19 symptoms, a Poisson model and a negative binomial model were used to fit and compare all analyses. In all cases, the negative binomial model provided the best fit. Logistic regression was used since the other outcome variable duration of COVID-19 symptoms is binary. After applying a Bonferroni correction to the 95% confidence intervals and *p*-values, p-values less than 0.001 are considered significant. A total of 14 measurements of psychological distress have been assessed in association with 2 COVID-19-related outcomes, resulting in 28 investigated associations.

## 3 Results

### 3.1 Descriptive statistics

Table 1 outlines the participants’ characteristics and the descriptive statistics of the measured variables. The correlation matrix (Table 2) shows that the different aspects of the DASS-21 scales are highly intercorrelated (a) within the same construct (i.e., depression, anxiety and stress), (b) within one time frame and (c) when measured at the same time point (i.e., the first survey).

**Table 1:** Descriptive statistics of all variables of interest.

Categorical variables	*n*	%
Male	1,218	40.3
Female	1807	59.7
Low	167	5.5
Mid	704	23.3
High	2,154	71.2
Smoker	201	6.6
COVID-19 Vaccinated	2,966	98.0
Previous COVID-19 infection	196	6.5
Use of Immunosuppressants	196	6.5
Use of vitamins or dietary supplements	1,041	34.4
Mental health problems	354	11.7
Use of Psychopharmaceuticals	402	13.3
> 5 days	1,085	35.9
> 10 days	399	13.2
0 days (asymptomatic)	186	6.2

Continuous variables	*M*	SD
Age	44.9	15.2
Comorbidities	0.1	0.3
BMI	25.4	4.7
Number of COVID-19 Symptoms	4.4	2.5
Depression medium-term before COVID-19	7.6	8.4
Depression short-term before COVID-19	5.3	7.9
Depression during COVID-19	6.4	7.7
Anxiety medium-term before COVID-19	5.0	6.1
Anxiety short-term before COVID-19	4.6	5.9
Anxiety during COVID-19	5.6	5.8
Stress medium-term before COVID-19	9.6	8.6
Stress short-term before COVID-19	6.4	8.0
Stress during COVID-19	7.0	7.9
Psychological Distress medium-term before COVID-19	22.2	20.7
Psychological Distress short-term before COVID-19	16.4	19.5
Psychological Distress during COVID-19	19.1	19.0

*N* = 3,025.

**Table 2:** Correlation matrix of all measurements of psychological distress.

Variable	1	2	3	4	5	6	7	8	9	10	11	12	13
(1) Use of psychopharmaceuticals	1												
(2) Mental Health Problems	0.327	1											
(3) Psychological Distress medium-term before COVID-19	0.299	0.332	1										
(4) Depression medium-term before COVID-19	0.260	0.288	0.882	1									
(5) Anxiety medium-term before COVID-19	0.263	0.282	0.791	0.576	1								
(6) Stress medium-term before COVID-19	0.278	0.323	0.933	0.732	0.666	1							
(7) Psychological Distress short-term before COVID-19	0.230	0.229	0.747	0.644	0.644	0.689	1						
(8) Depression short-term before COVID-19	0.194	0.208	0.637	0.667	0.474	0.543	0.854	1					
(9) Anxiety short-term before COVID-19	0.175	0.171	0.558	0.424	0.690	0.467	0.794	0.579	1				
(10) Stress short-term before COVID-19	0.219	0.211	0.711	0.572	0.549	0.735	0.886	0.671	0.574	1			
(11) Psychological Distress during COVID-19	0.222	0.230	0.662	0.574	0.560	0.612	0.676	0.569	0.545	0.619	1		
(12) Depression during COVID-19	0.196	0.191	0.567	0.573	0.432	0.488	0.585	0.581	0.425	0.504	0.870	1	
(13) Anxiety during COVID-19	0.200	0.199	0.503	0.389	0.558	0.440	0.541	0.404	0.587	0.448	0.787	0.559	1
(14) Stress during COVID-19	0.186	0.212	0.627	0.509	0.498	0.636	0.615	0.490	0.450	0.636	0.891	0.687	0.576

### 3.2 Main results

Results of all primary analyses appear in Table 3 (associations with number of COVID-19 symptoms) and 4 (associations with duration of COVID-19 symptoms). Long-term psychological distress prior to COVID-19 was assessed using two binary indicators rather than the DASS-21 to reduce potential recall bias associated with retrospective reporting over extended time frames. As a result, these findings are not directly comparable to those based on continuous measures of depression, anxiety, and stress, and are therefore presented in a separate subsection to ensure clarity and appropriate interpretation.

**Table 3:** Associations for number of COVID-19 symptoms using negative binomial regression.

Predictor	*β*	SE	*z*	*p*	corr *p*	IRR	Exp Diff	95% CL	
								LL	UL
Use of psychopharmaceuticals	0.1405	0.0280	5.0129	0.0000	0.0000	1.1509	0.6608	1.0462	1.2648
Mental health problems	0.1765	0.0291	6.0739	0.0000	0.0000	1.1930	0.8390	1.0807	1.3155
Psychological distress medium-term before COVID-19	0.1005	0.0098	10.2377	0.0000	0.0000	1.1057	0.4591	1.0693	1.1431
Depression medium-term before COVID-19	0.0719	0.0098	7.3392	0.0000	0.0000	1.0746	0.3285	1.0394	1.1108
Anxiety medium-term before COVID-19	0.1049	0.0095	11.0343	0.0000	0.0000	1.1106	0.4709	1.0750	1.1470
Stress medium-term before COVID-19	0.0934	0.0100	9.3494	0.0000	0.0000	1.0979	0.4328	1.0612	1.1357
Psychological distress short-term before COVID-19	0.1109	0.0092	12.0749	0.0000	0.0000	1.1173	0.5023	1.0828	1.1527
Depression short-term before COVID-19	0.0867	0.0092	9.4005	0.0000	0.0000	1.0906	0.3940	1.0568	1.1252
Anxiety short-term before COVID-19	0.1203	0.0090	13.3077	0.0000	0.0000	1.1278	0.5341	1.0935	1.1629
Stress short-term before COVID-19	0.0946	0.0095	10.0051	0.0000	0.0000	1.0992	0.4361	1.0643	1.1349
Psychological distress during COVID-19	0.1569	0.0088	17.7736	0.0000	0.0000	1.1698	0.7226	1.1350	1.2054
Depression during COVID-19	0.1254	0.0089	14.0575	0.0000	0.0000	1.1336	0.5788	1.0996	1.1685
Anxiety during COVID-19	0.1800	0.0085	21.0935	0.0000	0.0000	1.1972	0.8173	1.1627	1.2326
Stress during COVID-19	0.1229	0.0093	13.1707	0.0000	0.0000	1.1308	0.5725	1.0954	1.1671

*N* = 3,025.

Across all models, 27 out of 28 models were significant (*p* < 0.001) while considering the control variables and accounting for alpha inflation. In terms of overall psychological distress (depression, anxiety, and stress combined), psychological distress reported during COVID-19 tended to show the strongest effect, followed by short-term pre-infection distress, with weaker associations observed for more distal time frames. However, these differences in magnitude should be interpreted as descriptive patterns and trends, rather than definitive differences in effect size, since the confidence intervals overlapped between the parameter estimates. We did not find evidence of significant differences between magnitude of effect. When individual types of distress are compared, anxiety consistently showed the largest magnitude of effects in 5 out of 6 analyses, suggesting a stronger association with COVID-19 disease course compared to depression and stress, though not significantly different.

#### 3.2.1 Number of COVID-19 symptoms

Table 3 presents a more detailed overview of associations between all types of psychological distress in all time frames with the number of self-reported COVID-19 symptoms. The largest absolute point estimate for effect size is found for anxiety during COVID-19: an increase of one standard deviation in anxiety during COVID-19 corresponds with nearly 20 percent more symptoms. For the average person^1^ in this sample, this corresponds to an increase of 0.82 or almost one symptom. Of all the different measures of psychological distress before COVID-19 infection, anxiety short-term before COVID-19 shows the strongest association with the self-reported number of COVID-19 symptoms. Notably, these differences in magnitude are merely descriptive and based on absolute differences. There was not sufficient evidence to conclude that the magnitude of this effect was significantly greater than other effects, as the 95% confidence intervals consistently overlap within and across time frames.

##### 3.2.1.1 Long-term effects

The occurrence of mental health problems trended toward the strongest significant association: individuals with mental health problems have a nearly 20 percent higher rate of self-reported COVID-19 symptoms compared to those without mental health problems. However, the magnitude of this effect was not found to be significantly different from associations with the use of psychopharmaceuticals.

#### 3.2.2 Duration of COVID-19 symptoms

Table 4 presents an overview of the associations of all types of psychological distress (depression, anxiety, and stress) across time frames with the duration of the COVID-19 disease course. Results show similar trend to number of COVID-19 symptoms with anxiety during COVID-19 having the strongest absolute point estimate. A one standard deviation increase in anxiety during COVID-19 was associated with a 78.6% increase in the odds of symptom duration longer than 5 days. For an average person, this corresponds to an increase in the probability of experiencing symptoms lasting longer than 5 days by 12.1 percentage points. Although the effect of anxiety during COVID-19 was estimated to be strongest in magnitude, the magnitude of this effect was not found to differ significantly from the other types of distress (depression and stress) during COVID-19. The only significant difference in magnitude of effects was found between anxiety during COVID-19 and anxiety in the medium-term before COVID-19. The same pattern emerged for psychological distress before COVID-19. Anxiety short-term before COVID-19 shows the strongest magnitude point estimate with the duration of COVID-19 symptoms, though this difference in magnitude was not significantly different from other associations.

**Table 4:** Associations for duration of COVID-19 symptoms using logistic regression.

Predictor	*β*	SE	*z*	*p*	corr *p*	OR	Prob Diff	95% CL	
								LL	UL
Use of psychopharmaceuticals	0.3298	0.1114	2.9602	0.0031	0.1721	1.3907	0.0705	0.9515	2.0252
Mental health problems	0.5843	0.1169	4.9981	0.0000	0.0000	1.7937	0.1280	1.2069	2.6666
Psychological distress medium-term before COVID-19	0.2789	0.0407	6.8497	0.0000	0.0000	1.3217	0.0583	1.1522	1.5185
Depression medium-term before COVID-19	0.2086	0.0396	5.2639	0.0000	0.0000	1.2319	0.0436	1.0775	1.4095
Anxiety medium-term before COVID-19	0.2483	0.0403	6.1612	0.0000	0.0000	1.2818	0.0510	1.1191	1.4707
Stress medium-term before COVID-19	0.2788	0.0406	6.8643	0.0000	0.0000	1.3215	0.0593	1.1523	1.5174
Psychological distress short-term before COVID-19	0.3294	0.0401	8.2107	0.0000	0.0000	1.3901	0.0683	1.2151	1.5949
Depression short-term before COVID-19	0.2721	0.0389	6.9920	0.0000	0.0000	1.3128	0.0566	1.1517	1.4996
Anxiety short-term before COVID-19	0.3375	0.0406	8.3051	0.0000	0.0000	1.4014	0.0683	1.2232	1.6112
Stress short-term before COVID-19	0.2729	0.0395	6.9065	0.0000	0.0000	1.3138	0.0577	1.1500	1.5032
Psychological distress during COVID-19	0.5273	0.0420	12.5434	0.0000	0.0000	1.6944	0.1124	1.4727	1.9581
Depression during COVID-19	0.4456	0.0404	11.0239	0.0000	0.0000	1.5614	0.0952	1.3642	1.7942
Anxiety during COVID-19	0.5798	0.0435	13.3222	0.0000	0.0000	1.7857	0.1214	1.5450	2.0748
Stress during COVID-19	0.3917	0.0403	9.7302	0.0000	0.0000	1.4795	0.0842	1.2925	1.6978

*N* = 3,025.

##### 3.2.2.1 Long-term effects

When examining psychological distress long-term before COVID-19, we found that experiencing mental health–related work absence was associated with a 2.16-fold increase in the odds of symptom duration longer than 5 days. For an average person, this corresponds to an increase in the probability of symptoms lasting longer than 5 days by almost 13 percent. We did not find evidence that the use of psychopharmaceuticals was significantly associated with the duration of COVID-19 symptoms.

#### 3.2.3 Control variables

In separate analyses, generalized linear models with only the control variables as predictors were fitted for all outcomes. Interested readers can find the estimated coefficients for all control variables, including uncorrected *p*-values, in Supplementary materials. For all outcomes, women reported more severe COVID-19 disease courses than men. No evidence for effects of vaccination status, comorbidities, use of immunosuppressive medications, educational level, and smoking were found.

## 4 Discussion

This study aimed to explore (1) the associations between different types of psychological distress and the COVID-19 disease course, and subsequently explore (2) which type of psychological distress (depression, anxiety, stress) is associated with adverse COVID-19 outcomes, and (3) whether the time frame (long-term before, medium-term before, short-term before, and during COVID-19) might influence these outcomes. This study contributes to a growing body of literature indicating a role for psychological distress in the COVID-19 disease course. We found that all measured forms of psychological distress (depression, anxiety, and stress) were independently associated with COVID-19 outcomes, and these associations were observed regardless of the timing of psychological distress exposure.

Our findings confirm previous evidence demonstrating the negative impact of psychological distress on COVID-19 disease course in outpatients, emphasizing a psychoneuroimmunological pathway underlying COVID-19 (Pływaczewska-Jakubowska et al., 2022; Ayling et al., 2022). We observed that all types of psychological distress before and during COVID-19 were significantly associated with adverse COVID-19 outcomes, except for the association between the use of psychopharmaceuticals 3 years before COVID-19 and duration of symptoms. While the effects observed in our study are small, this is not uncommon in research investigating complex and dynamic phenomena. Multiple factors play a role in the relationship between psychological distress and the disease course of a viral infection. Therefore, when significant associations are found we should carefully consider potential consequences without overestimating the relative impact of the factors involved.

Regarding the *type of psychological distress*, we observed that all forms of psychological distress (depression, anxiety, and stress) showed a significant impact on COVID-19 disease course (number and duration of symptoms), with anxiety trending toward the strongest associations. Although anxiety showed the largest absolute magnitude of effects, the magnitude was not significantly different from the effect of depression or stress. Across time frames, we observed a consistent trend, with anxiety showing the highest point estimate, suggesting that anxiety may play an important role in the course of COVID-19. Earlier, Koyama et al. (2022) also found that anxiety was related to adverse COVID-19 outcomes in hospitalized patients. The present findings extend this association to home-isolated, ambulatory individuals, suggesting that the relationship between psychological distress and COVID-19 disease course is not limited to severe or inpatient populations.

Regarding the *timing of psychological distress*, our findings were somewhat unexpected. Based on previous studies, we expected that the longer psychological distress was present, the stronger the association between psychological distress and adverse COVID-19 outcomes would be (Segerstrom and Miller, 2004). However, we found that psychological distress during COVID-19 trended toward the strongest association with both COVID-19 outcomes (although the differences in effects were not significant). When focusing solely on the time frame of the experienced psychological distress before infection, we found the largest magnitude of effect between psychological distress short-term (i.e., during 1 week) before COVID-19 infection and adverse COVID-19 outcomes, compared to psychological distress experienced medium-term (i.e., during 1 year) before the infection. These differences were not statistically significant and represent a notable trend.

Interpretation of effect magnitude in this study should be approached with caution. Differences in effect size did not reach statistical significance, the retrospective design introduces potential recall bias, and observed effect sizes were modest. However, the consistency of associations across psychological distress domains and time frames suggests a potentially robust relationship that warrants further investigation. In the present study, differences in the strength of associations across time frames were generally not statistically significant. This might reflect a genuine lack of difference in the strength of associations, may reflect low power to detect differences in magnitude of effects, or could be due to the high intercorrelations among the different DASS-21 scales (which reduce the likelihood of detecting significant differences in their correlations with other variables). These intercorrelations are partially artificial (e.g., because all scales load highly on negative affectivity), a common characteristic of self-report questionnaires assessing psychological distress. Therefore, it may be valuable to replicate this study using alternative measures.

Previous research found that people with pre-existing mental disorders and people who experienced long-term distress, such as burnout and sleep problems, are more likely to experience worse COVID-19 outcomes (Kim et al., 2021; Ayling et al., 2022; Fond et al., 2021; Wang et al., 2021). These findings are only partially confirmed in our study. Mental health problems, as an indicator of long-term psychological distress, show significant associations with all examined COVID-19 outcomes. Conversely, the use of psychopharmaceuticals, as another indicator of long-term psychological distress, was not found to be significantly associated with the duration of COVID-19 symptoms. In this regard, it is noteworthy that Zheng et al. (2022) demonstrated that the use of some psychopharmaceuticals, antidepressants (SSRIs) for instance, can have positive effects on the COVID-19 disease course. This suggests that different types of psychotropic medication may have divergent effects, which our binary measure could not capture. Future research should expand the research using more nuanced measures to differentiate between the types of psychopharmaceuticals used to investigate the complex relationships with viral infections’ disease course.

Although we cannot draw conclusions about the directionality of the observed associations in this study, findings contribute to a substantial body of longitudinal and experimental research suggests that psychological stress can negatively affect the immune system and thereby influence the course of viral infections, including COVID-19 (see, e.g., Cohen et al., 2007; Goodwin et al., 2014; O’Connor et al., 2021; Segerstrom and Miller, 2004). In light of these findings, it is important to consider the potential sources of psychological distress during the past COVID-19 crisis. One factor that has received considerable attention is the use of fear-based messaging in government communication and media coverage. While the associations between fear messaging, psychological distress, and COVID-19 outcomes were not directly examined in this study, our findings underscore the relevance of this debate. Concerns raised by several scholars during the COVID-19 crisis regarding the widespread use of fear appeals in public health communication and news media coverage may merit further examination (see, e.g., Asmundson and Taylor, 2020; Swami and Gupta, 2021; Stolow et al., 2020; Warren et al., 2024). The use of fear appeals is a highly debated topic that raises ethical questions (Tannenbaum et al., 2015). For instance, if fear is assumed to be a powerful motivator for behavior while also potentially posing health risks, the use of fear appeals to achieve a desired public health outcome becomes a complex ethical consideration – one that only can be assessed in the light of the severity of an outbreak at the time it occurs. Further interdisciplinary dialog and inquiry is desirable on this important topic.

In addition, our findings support the large body of evidence showing a relationship between mental health and viral outcomes, highlighting that human health involves complex interactions between biological, psychological, and social factors, which is also relevant in the context of viral infections like SARS-CoV-2. As pointed out by several scholars, a greater recognition and understanding of the biopsychosocial model of health is necessary to be better prepared for future health crises (see, e.g., Claes et al., 2023; Jones et al., 2023). This implies acknowledging that body and mind are interconnected in a non-linear way, unfolding dynamically across time and developing within a social context. It is desirable that policy makers consider all aspects of health and integrate mental health support into public health responses.

Each person experienced unique potential psychosocial stressors before and during the COVID-19 pandemic, and each reacts to psychological distress in a distinct and personal way. Future research should explore whether there are certain groups, e.g., distinguished by personality style, socioeconomic background, or age, who are particularly vulnerable to the risk of particular psychological distress experiences impacting viral disease course. In addition, research into protective factors, such as coping strategies, emotional regulation, social support, hope, self-efficacy, health behavior patterns, and spiritual and existential grounding, may help explain why some individuals are more psychologically resilient than others. These protective factors can reduce physiological stress responses and strengthen the immune system, thereby promoting recovery, which may lead to better COVID-19 outcomes (Abate et al., 2024; Cohen et al., 1997; Uchino, 2006).

This awareness is essential when making political choices during health crises and under normal circumstances, particularly in this post-pandemic era. Previous epidemics and traumatic global events (i.e., SARS, 9/11) have shown that the mental health effects of these events might persist for many years (Turna et al., 2021). Our findings add to the growing body of evidence, including experimental research, suggesting that stress and anxiety may modulate immune response, and therefore opens potential pathways for modifiable points of intervention (Alawna and Mohamed, 2022; Dutcher et al., 2022; Witek Janusek et al., 2019; Schakel et al., 2019).

### 4.1 Strengths and limitations

Our study has notable strengths. First, we studied a sizeable sample of the Flemish population consisting of non-hospitalized COVID-19 patients with a confirmed PCR test for COVID-19. Second, data were collected during the COVID-19 pandemic at two time points, which enabled us to measure psychological distress both before and during the COVID-19 infection. Third, many relevant variables concerning the disease course of COVID-19 were collected and included as control variables. Fourth, to our knowledge, this is the first study examining the differential impact of different types of psychological distress at different time frames within one study sample. We provide further insight into the complex interaction between psychological distress and COVID-19 disease outcomes. We have provided a clear call for future research.

Admittedly, our study has limitations. First, it relies entirely on self-reported data, rendering it susceptible to social desirability and retrospective bias concerns. Participants with severe COVID-19 may have been inclined to report higher levels of psychological distress during their illness, seeking causal frameworks to help them understand why they became so ill. Furthermore, psychological distress can lead to the overreporting symptoms. Recall bias may have been present as there was a time lapse between the disease course and completing the survey. Specifically, retrospective accounts of psychological distress may be biased by issues of memory encoding and retrieval under conditions of stress. We mitigated against recall bias in long-term report by including two binary items that were designed to be less susceptible to recall bias and subjective interpretation; however, use of single items has its own measurement limitations. The continuous DASS-21 scores used for the other time frames are not directly comparable to this binary measure. A second limitation is that our statistical analysis reports regression analysis based on retrospective observational data, which cannot establish causal connections. However, interventional experiments on this topic are ethically problematic. Furthermore, causality may run in both directions: Psychological distress makes it harder to recover from a viral infection, but conversely, being ill can cause feelings of psychological distress as well. The potential for bidirectionality was mitigated by assessing psychological distress at time frames relative to illness onset; however because data were collected concurrently, retrospective reporting may have introduced recall bias, temporal ambiguity, and common method variance, limiting causal interpretation. We additionally acknowledge the collinearity issues between different types of psychological distress. Although this overlap was accounted for in the analytic approach, future research is needed to confirm the magnitude and stability of the observed associations. Third, adapted time anchors were used for the DASS-21 to obtain a measure of various time frames using the same item set. Future studies should examine potential measurement noninvariance of the scale across these timeframes. Additionally, we could not obtain DASS-21 scores long-term before the COVID-19 infection (i.e., before the onset of the COVID-19 pandemic) due to measurement bias associated with distal recall. Fourth, we could not obtain biological samples such as levels of pro-inflammatory cytokines or cortisol or gather information about measured viral load, so we could not differentiate between different SARS-CoV-2 variants. The purpose of this study was to observe the association between time of psychological stress and COVID-19 outcomes; future work should explore the differential mechanisms through which long-term versus concurrent psychological distress increases susceptibility and severity of disease. Fifth, it is hard to determine the exact time point of COVID-19 infection since respondents tested for it at various points in the disease course. Finally, adults who did not have access to email and the internet could not participate.

## 5 Conclusion

All types of psychological distress experienced at different time frames were significantly associated with adverse COVID-19 outcomes, except for the association between psychopharmaceutical use 3 years before COVID-19 infection and duration of symptoms. The type and the time frame of the psychological distress seem to influence these associations: psychological distress, particularly anxiety, experienced short-term before and during COVID-19 infection, tended to show the strongest associations with a worse COVID-19 disease course, though not significantly different. Although causal inferences cannot be made, these findings highlight the potential relevance of psychological distress in the context of infectious disease, emphasizing the importance of considering psychological conditions associated with public health crises.

## Data Availability

The raw data supporting the conclusions of this article will be made available by the authors, without undue reservation.

## Footnotes

1 The hypothetical average person has an average age, BMI, distress level, and number of comorbidities, and represents the statistical mean across gender, previous infection, use of corticosteroids or immunosuppressants, use of vitamins and dietary supplements, COVID-19 vaccination, and smoking. For education level, which had three categories, this person corresponds to the middle category.
