# Supplementary material for: Depression, anxiety, and stress at different time frames in relation to COVID-19 disease course: a Flemish observational study
Source: Front Psychol. 2026 Oct 20;17:1765017. doi: 10.3389/fpsyg.2026.1765017 (PMC13727632; doi:10.3389/fpsyg.2026.1765017)
Supplement: Supplementary file 1 [file Supplementary_file_1.docx]

Supplementary Material

# 1 Supplementary Figures and Tables

Table S1

Chronbach’s Alpha Values for DASS-21 scales across different Time Frames

Table S2

Associations for Duration of COVID-19 Symptoms using Logistic Regression (cut-off point at 0 days – asymptomatic cases)

Note. N=3025

Table S3

Associations for Duration of COVID-19 Symptoms using Logistic Regression (cut-off point at 10 days)

Note. N=3025

Table S4

Associations for Number of COVID-19 Symptoms using Negative Binomial Regression

Note. N=3025; This model includes only the control variables and is not corrected for multiple testing.

Table S5

Associations for Duration of COVID-19 Symptoms using Logistic Regression

Note. N=3025; This model includes only the control variables and is not corrected for multiple testing.
